# There Was Something in the Air: A Case of Interstitial Lung Disease Associated With Continuous Positive Airway Pressure (CPAP) Foam Degradation and Inhalation Syndrome

**DOI:** 10.7759/cureus.82595

**Published:** 2025-04-19

**Authors:** Ma D Valdes Bracamontes, Gangacharan Dubey

**Affiliations:** 1 Pulmonary and Critical Care, SUNY (State University of New York) Downstate Health Sciences University, Brooklyn, USA; 2 Pulmonary and Critical Care Medicine, VA NY (Veterans Affairs New York) Harbor Healthcare System, Brooklyn, USA

**Keywords:** continuous positive airway pressure (cpap), interstitial lung disease (ild), obstructive sleep apnea (osa), polyurethane foam degradation, veterans affairs (va)

## Abstract

Polyurethane foam degradation in ventilatory support devices led to a recall by Philips Respironics after users developed respiratory symptoms and noted a sediment in the tubing system of such devices. Foam degradation releases end products that have an irritative effect on the airway. Although the incidence of neoplastic processes did not increase among users, studies for other conditions are scant. The following case presents the association between the use of one of these devices and a new diagnosis of interstitial lung disease, which resolved upon discontinuation of the device, in a patient with obstructive sleep apnea and chronic obstructive pulmonary disease.

## Introduction

From production, operating life, until disposal, plastic products in the environment find a way to different locations within our bodies, including the lungs. On June 14, 2021, Philips Respironics issued a device recall for appliances used to provide both non-invasive and invasive mechanical ventilatory support. These are continuous positive airway pressure (CPAP), bi-level PAP, and continuous ventilators. The use of these devices is not limited to healthcare facilities, they can also be found in different places such as homes, nursing homes, skilled nursing facilities, and hospitals. They play an important role in the management of different respiratory conditions. 

Within, they contain a sound abatement polyurethane foam (PUF) which degrades with time, releasing particles and gases that irritate the airway. Users of these devices developed respiratory symptoms and noted a sediment in the tubing from the device to the mask. Although cytotoxic, cancer incidence did not increase among users. Studies for other conditions are scant. The following case presents the association between PUF-CPAP use and unexpected outcome in a patient with obstructive sleep apnea (OSA) and chronic obstructive pulmonary disease (COPD).

From June 2021 to August 2022, the U.S. Veterans Health Administration faced a manufacturer’s recall of devices. A polyester-based polyurethane sound abatement foam in old devices, poorly stored [1,2], degrades into irritative particles and gases that can be inhaled. On July 8, 2021, Philips informed that the by-products are cyto and genotoxic [2-4]. Exposure during plastics manufacture, processing, or recycling injures the lungs. Dust and fume inhalation can lead to an interstitial lung disease (ILD), a group of conditions damaging the lung interstitium, the space formed by the fluid and the tissue that connects and provides support to the alveolar sacs and airway, maintaining a cohesive lung architecture. Often progressive, these conditions may develop from extracellular components building up inflammation with thickening and stiffening of the interstitium, impairing breathing, followed by fibrosis. In our case, resolution ensued after device discontinuation.

## Case presentation

A 38-year-old male presented to the pulmonary clinic at a VA Hospital with progressively worsening exertional dyspnea of several years’ duration. His comorbidities included COPD with a 40-pack-year smoking history and OSA treated with CPAP. He had an occupational history of three years peeling off wall paint since the age of 18. He had an exercise tolerance of half a block. Previous pulmonary function test (PFT) and computerized tomography (CT) chest showed emphysema and lung nodules with normal diffusing capacity of the lungs for carbon monoxide (DLCO).

**Figure 1:**
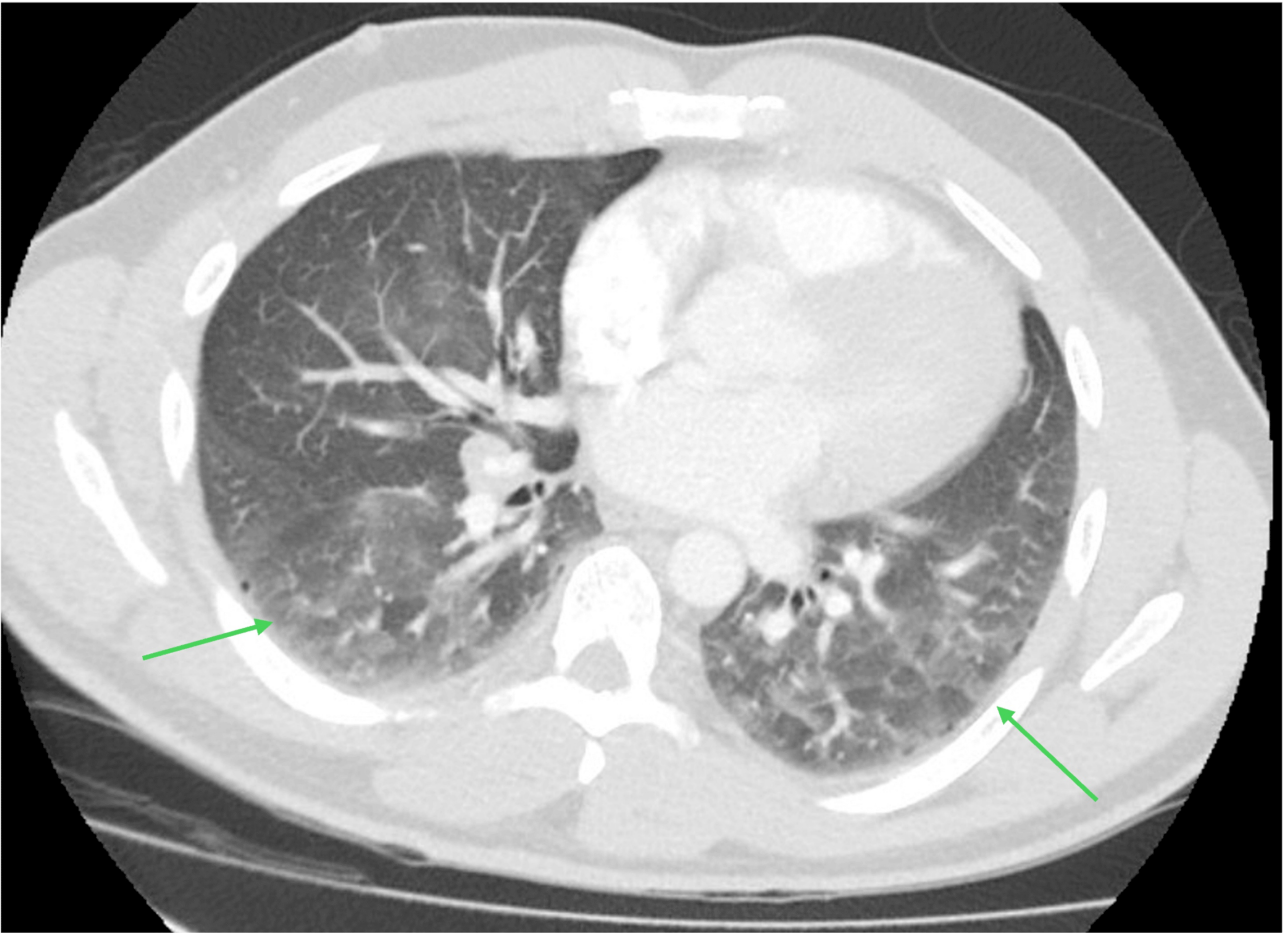
CTA showing ground-glass opacities in the lower lobes CTA, computerized tomography angiogram.

CT angiogram (CTA) revealed mediastinal adenopathy, centrilobular and paraseptal emphysema, and moderate ground-glass opacities (GGO) in the lower lobes (Figure 1). Repeat PFT showed a moderate gas transfer defect, explaining his dyspnea and possible ILD. Our plan was to obtain serial CT chest and PFT, and if the abnormalities persist or worsen, to obtain video-assisted thoracic surgery lung biopsy.

Then, the patient received a letter from the Veterans Administration on June 30, 2021, informing him of the issues with foam degradation in the CPAP machine, and he was advised to stop using the CPAP machine, which he did.

**Figure 2:**
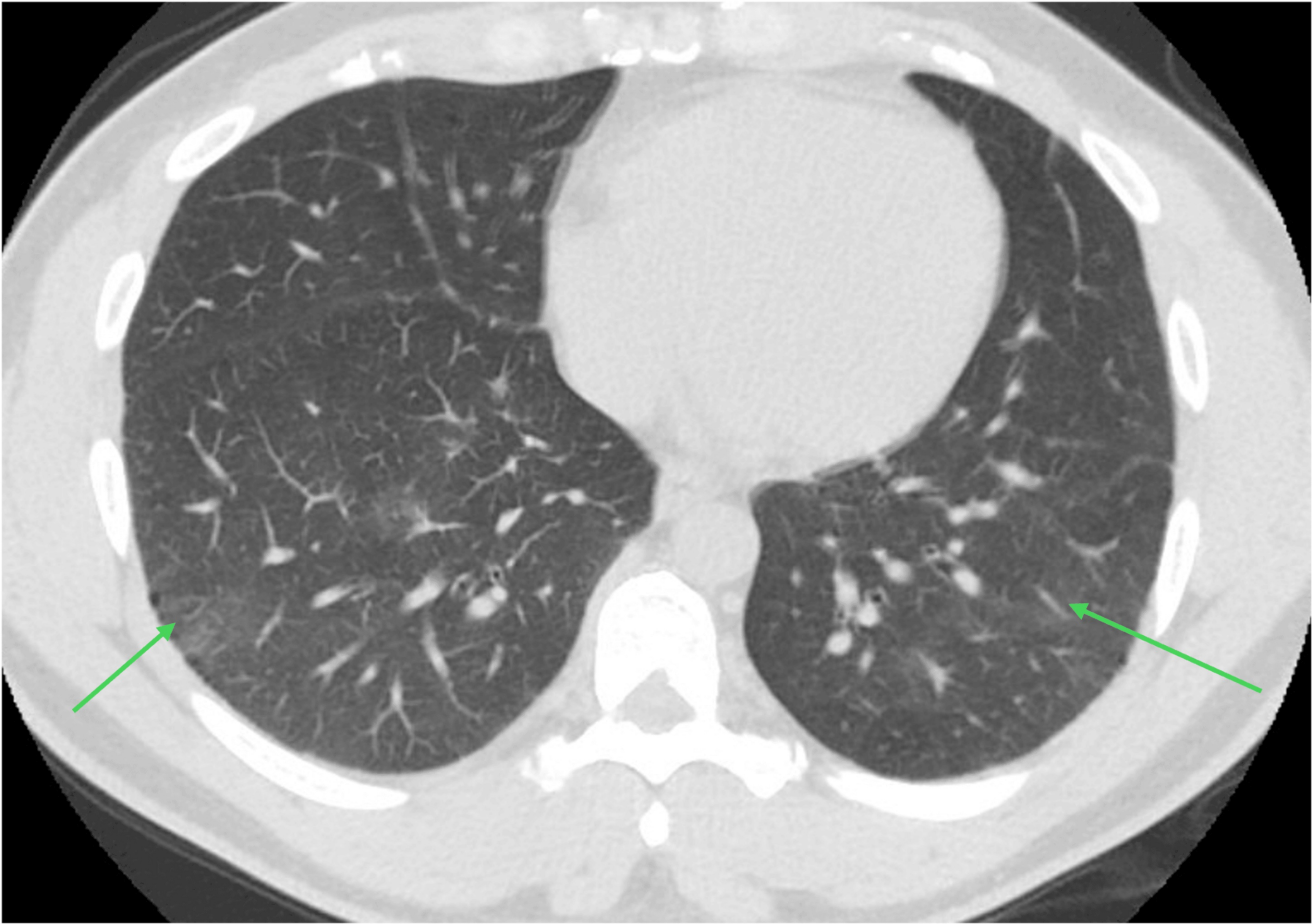
CT chest taken in August 2021, three months after the patient stopped using the CPAP machine, showed an interval decrease with mild residual GGO CPAP, continuous positive airway pressure; GGO, ground-glass opacities.

**Figure 3:**
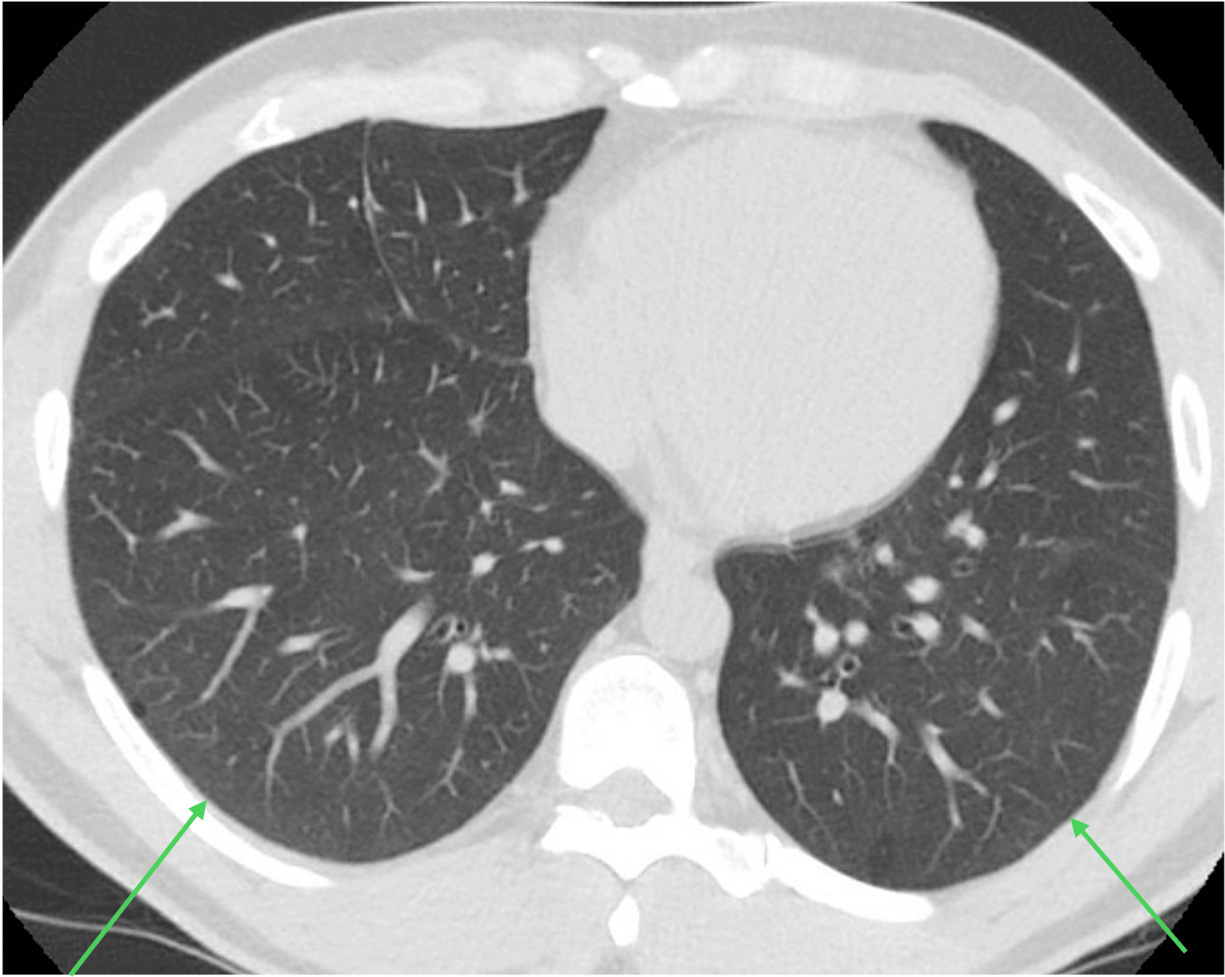
CT at eight months showed an interval resolution of previously noted GGO in both lungs GGO, ground-glass opacities.

Serial CT chest showed mild residual GGO (Figure 2), followed by complete radiological resolution (Figure 3). Repeat PFT showed mild obstruction, but the gas transfer defect completely normalized. On further follow-up visits, the patient’s exertional dyspnea was also resolved.

DLCO in 2020 was 53% when the patient was using a Philips Respironics CPAP machine for his OSA, and later in 2022, after he discontinued using the CPAP machine in June 2021, his DLCO normalized to 77% (Table 1).

**Table 1:** PFT comparison during the use of CPAP and after its discontinuation. PFT after CPAP discontinuation was performed after the latest update of the ATS guidelines for PFT interpretation. PFT: pulmonary function test; CPAP: continuous positive airway pressure; ATS: American Thoracic Society; BD: bronchodilator; FVC: forced vital capacity; FEV1: forced expiratory volume in the first second; VC: vital capacity; TLC: total lung capacity; ERV: expiratory reserve volume; RV: residual volume; MVV: maximal voluntary ventilation; DLCO: diffusing capacity of the lungs for carbon monoxide.

PFT	After CPAP discontinuation	While using CPAP
Spirometry		
FVC	Pre-BD: 5.06 L (96%); post-BD: 4.92 L (93%)	Pre-BD: 4.91 L (92%); post-BD: 5.28 L (99%)
FEV_1_	Pre-BD: 3.50 L (82%); post-BD: 3.54 L (83%)	Pre-BD: 3.63 L (84%); post-BD: 3.91 L (90%)
FEV_1_/FVC	72 (LLN: 81)	74 (LLN: 81)
Significant bronchodilator response	No	No
Lung volumes		
VC	5.16 L (97%)	4.91 L (92%)
TLC	7.57 L (107%)	6.84 L (96%)
ERV	1.27 L (73%)	1.40 L (79%)
RV	2.41 L (129%)	1.93 L (105%)
Diffusing capacity		
DLCO	22.1 mL/mmHg/min (79%)	22.2 mL/mmHg/min (55%)
DLCO adjusted	21.6 mL/mmHg/min (77%)	21.5 mL/mmHg/min (53%)
Impression	There is mild obstruction, with no significant bronchodilator response. There is no restriction, with air trapping, and hyperinflation. There is no gas transfer defect	There is no evidence of obstruction, significant bronchodilator response, restriction, hyperinflation, or air trapping. There is moderate gas transfer defect

## Discussion

In the past few decades, micro- and nanoplastics (MNPs) have been identified as widespread environmental contaminants, and research has emphasized the dreadful impact they have on respiratory organs among different species worldwide. Once they are found in the human lung tissues, a concern raises about their potential role in damaging lung parenchyma’s architecture, and their contribution in the development of ILD by leading to a chronic state of inflammation, building up within the lungs, triggering immunological reactions, and tissue injury [2,3,5-9] with subsequent scarring of lung tissues secondary to changes in the extracellular matrix. The hazard from PUF-CPAP devices remarks the need for studies and clinical trials. The case described above exemplifies the development of a complex inflammatory ILD in a susceptible patient who was exposed to an inhaled plastic antigen. Due to the heterogeneity and overlap among the different ILDs, diagnosis and treatment are challenging. A high index of suspicion is required and should be on the differential diagnosis when a patient presents with a clinical picture and imaging suggestive of ILD. Recognizing this case as inhalation lung injury and more specifically associated with foam exposure was only possible during the follow-up clinic visits with serial images upon discontinuation of CPAP machine use, with subsequent resolution of the condition, both clinically and radiographically. There is no experimental data simulating the PUF-CPAP user pattern [1,5]. The DISCOVERY cohort (DISease in patients reported to the Swedish CPAP Oxygen and VEntilator RegistrY) studied the risk factors for incidence, disease course, quality of life, hospitalization, and mortality in the cases of chronic respiratory failure and OSA on CPAP [4-6]. Lung injury from MNP inhalation is evident in occupational exposures [1,4]. But, this hazard coming from several devices designed for the management of diverse medical conditions was brought to light after the Philips Respironics incident [6]. Due to the potential carcinogenic and airway irritative effects of polyester-based polyurethane contained in the sound-absorbing foam of positive airway pressure devices, its continuous use raises many concerns. However, despite the need for more anti-obstructive drugs, patients using the devices did not have increased risk of lung cancer [3,4,6-10], but experienced interstitial inflammation and pulmonary fibrosis [1,7-10].

## Conclusions

It is unclear to what extent lung damage occurs upon MNP exposure. Trials for informed clinical decision-making are needed. Association between CPAP use, worsening symptoms, gas transfer defect on PFT, and imaging findings on presentation, followed by clinical improvement, absence of gas transfer defect, and imaging resolution upon device discontinuation led to believe the initial CT findings that represent foam inhalation lung injury, which resolved a month after the device use was stopped. When comparing both sets of PFT, it is important to note that the patient had a 40-pack-year smoking history and occupational exposure when peeling off paint, and both of them pose the risk for the development of pulmonary disease. Smoking is the strongest risk factor for developing COPD. COPD is associated with an obstructive pattern and hyperinflation in the repeat PFT, and these findings are the opposite of a restrictive pattern. A restrictive pattern associated with a gas transfer defect is an expected finding in ILD or pulmonary fibrosis. Gas transfer defect resolved in the repeat PFT, which supports the association between CPAP use and foam inhalation; after discontinuing exposure to the insulting agent (foam coming from the CPAP device), the diffusion defect resolved. There were no similar cases of ILD associated with the use of Phillips Respironics CPAP found in our literature review done at the time of this report. This case report emphasizes the need for research, safety strategies for medical device use, and prompt intervention after a critical incident such as this recall. 
